# The structural properties of full-length annexin A11

**DOI:** 10.3389/fmolb.2024.1347741

**Published:** 2024-03-07

**Authors:** Erika F. Dudas, Mark D. Tully, Tamas Foldes, Geoff Kelly, Gian Gaetano Tartaglia, Annalisa Pastore

**Affiliations:** ^1^ Dementia Research Institute at King’s College London, The Wohl Institute, London, United Kingdom; ^2^ European Synchrotron Radiation Facility, Grenoble, France; ^3^ University College London, Department of Physics and Astronomy, University College London, London, United Kingdom; ^4^ Institut de Biologie Structurale (IBS), Institut Laue-Langevin, University Grenoble Alpes, Grenoble, France; ^5^ MRC Biomedical NMR Centre, The Francis Crick Institute, London, United Kingdom; ^6^ Italian Institute of Technology, CHT@Erzelli, Genova, Italy

**Keywords:** amyotrophic lateral sclerosis, annexins, intrinsically unstructured regions, NMR, small angle X-ray scattering, structure

## Abstract

Annexin A11 (ANXA11) is a calcium-dependent phospholipid-binding protein belonging to the annexin protein family and implicated in the neurodegenerative amyotrophic lateral sclerosis. Structurally, ANXA11 contains a conserved calcium-binding C-terminal domain common to all annexins and a putative intrinsically unfolded N-terminus specific for ANXA11. Little is known about the structure and functions of this region of the protein. By analogy with annexin A1, it was suggested that residues 38 to 59 within the ANXA11 N-terminus could form a helical region that would be involved in interactions. Interestingly, this region contains residues that, when mutated, may lead to clinical manifestations. In the present study, we have studied the structural features of the full-length protein with special attention to the N-terminal region using a combination of biophysical techniques which include nuclear magnetic resonance and small angle X-ray scattering. We show that the N-terminus is intrinsically disordered and that the overall features of the protein are not markedly affected by the presence of calcium. We also analyzed the 38–59 helix hypothesis using synthetic peptides spanning both the wild-type sequence and clinically relevant mutations. We show that the peptides have a remarkable character typical of a native helix and that mutations do not alter the behaviour suggesting that they are required for interactions rather than being structurally important. Our work paves the way to a more thorough understanding of the ANXA11 functions.

## Introduction

The length of neurons, the main components of the nervous system, can range from less than a millimeter to over a meter in some cases. In some animals, such as certain species of whales, axons can be up to 30 m in length. However, whatever their lengths, neurons must sense and respond to stimuli and readily transfer signals to all their regions in the order of milliseconds. To fulfil this need, neurons rely on specialized machines that permit the synthesis of proteins locally and deliver mRNAs from the cell body to the different remote locations (Fernandopulle et al., 2019). An elegantly regulated way to transport mRNA involves the formation of discrete RNA granules (Redpath and Ananthanarayanan, 2023), which are membraneless organelles that contain both RNA and RNA-binding protein aggregates (Hyman and Brangwynne, 2011; Hyman et al., 2014). Organelle transport inside the cells usually requires both the molecular motor proteins kinesin and dynein and microtubules that provide polarized tracks which, depending on the molecular motor, allow movement from/to the dendrites and the axon (Cason and Holzbaur, 2022). Any impairment of this information leads to neurodegenerative diseases, including Alzheimer disease and amyotrophic lateral sclerosis (ALS) (Fallini et al., 2016; Brown and Al-Chalabi, 2017).

Recently, a novel mechanism, termed “hitchhiking,” was described, in which organelles can traffic along the microtubules not by directly interacting with the motors but by temporarily “hitchhiking” being bound to other organelles that are already moving and that act as “vehicles” to support the movement of other cargos (Salogiannis and Reck-Peterson, 2017). This mechanism needs other molecules to act as a tether. One of the proteins that has been described as a molecular tether between RNA granules and lysosomes is annexin A11 (ANXA11) (Fernandopulle et al., 2021). ANXA11 has also been linked to amyotrophic lateral sclerosis (ALS), an incurable progressive motor neuron disease. ALS has been associated to many different genes that encode RNA-binding proteins, mostly involved in RNA trafficking, that, when mutated, can lead to irreversible protein aggregation and disease (Brown and Al-Chalabi, 2017; Fernandopulle et al., 2019; Zaepfel and Rothstein, 2021).

**SCHEME 1:**
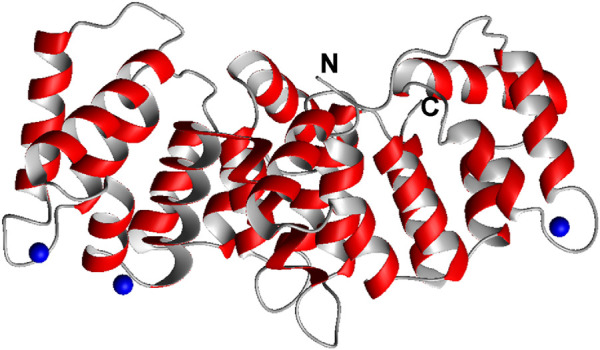
Crystal structure of the partially Ca^2+^ loaded C-terminal core domain of ANXA11 (6tu2, residues 188–-503). The N and C termini are indicated. Ca^2+^ ions are shown in blue. In other annexins each repeat can bind up to 2 Ca^2+^ ions. Shown is only one monomer of the three in the asymmetric unit. Sequence of the proline-rich N-terminal domain.

ANXA11 is a 56 kDa widely expressed protein, that belongs to the annexin protein family whose members play an important role in cell division, calcium signalling, vesicle trafficking and apoptosis (Vedeler and Hollås, 2000; Filipenko et al., 2004; Vedeler et al., 2012; Bharadwaj et al., 2013). Annexins are calcium-dependent proteins whose primary function is binding to phospholipids. In a recent elegant work, it was also conclusively shown that ANXA11 binds to RNA and that RNA-binding seems to be a common feature of the whole annexin family, suggesting a general role of these proteins in granule trafficking (Patil et al., 2023). The annexin structure comprises a conserved C-terminal core domain that is formed by four helical repeats (annexin repeats) well distinct from other calcium-binding motifs (Scheme 1 and Supplementary Figure S1). Each annexin motif contains ∼70 amino acids and is arranged into five α-helices, termed A–E (Gerke and Moss, 2002). The loops connecting the AB and DE helical hairpins contain the Ca^2+^ binding sites, with helix C packed against the other components of the bundle orthogonally. Lipid binding involves the core domain and seems to be coupled, at least in some members of the family, to a conformational change induced by Ca^2+^ binding (Gerke et al., 2005). The core domain is preceded by a highly variable region both in sequence and N-terminus length that is thought to mediate interactions. The recent paper by the Vedeler’s group demonstrates that RNA-binding is mostly contributed by the C-terminus of ANXA11 although we do not know where or how, whereas the N-terminus has some minor role in modulating the interaction (Patil et al., 2023).

The structure of the C-terminal domain of ANXA11 has been solved (Lillebostad et al., 2020) and, as expected, superposes with the corresponding region of other annexins within 1.2 Å. The N-terminal domain of ANXA11 is ∼200 residues (one of the longer in the annexin family) and contains low complexity regions dominated by prolines which account for 1/3 of the residues. Several mutations were identified in a thorough screening of a large cohort of familial ALS patients in the non-conserved N-terminus, including the p.D40G and p.G38R variants in the N-terminus (Smith et al., 2017; Teyssou et al., 2021). Although unique to ANXA11, the N-terminus contains a motif that is reminiscent of the N-terminus of annexin A1 (ANXA1) (de Souza Ferreira et al., 2023). In this protein, residues 2–26 form a helix and fold back to pack against the core domain in the absence of calcium. Calcium binding causes a conformational rearrangement of the core domain and the release of the helix which becomes available for interactions with other proteins (Réty et al., 2000; Rosengarth et al., 2001; Rosengarth and Luecke, 2003). A similar mechanism of regulation was suggested for ANXA11, and a putative helical motif was identified around residues 38–59 (Smith et al., 2017). It was also suggested that regulation of interactions with the apoptosis-linked gene-2 protein (ALG-2) and S100A6 (calcyclin) (Rintala-Dempsey et al., 2008) occurs through a Ca^2+^-induced conformational rearrangement of the C-terminus that leads to release of the N-terminus, making it proficient for interaction with its partners (Smith et al., 2017). These two interacting proteins seem to be potent regulators of ANXA11-based liquid-liquid phase separation which affects formation of ribonuclear granules (Nixon-Abell et al., 2023). This phenomenon is thought to be at the very basis of mRNA transport in neurons (Brangwynne et al., 2009; Kato and McKnight, 2018). Accordingly, a ANXA11 p.D40G ALS-related mutation was proven experimentally to abolish calcyclin binding (Smith et al., 2017), whereas no effect was observed with a p.G38R mutant. Despite this evidence, definite validation of the 38–59 helical hypothesis may only be achieved by solving the structure of full-length ANXA11.

In the present study, we used a hybrid approach based on a combination of spectroscopic methods and small-angle X-ray scattering (SAXS) to characterize the structure of full-length ANXA11. We proved that the N-terminal domain is intrinsically disordered and determined its relative orientation as compared to the C-terminal core domain in a calcium-dependent manner. We then structurally characterized synthetic peptides encompassing the sequence of the region 38–59 of wild-type and mutated ANXA11 and proved that they both adopt a helical structure. Finally, we used advanced computational tools (Bolognesi et al., 2016; Livi et al., 2016) to predict regions with RNA-binding properties and granule-forming tendencies. Our evidence fully supports the helix hypothesis and suggests a distinct and specific role of the N-terminal domain of ANXA11 in its tethering functions.

## Materials and methods

### Sample preparation

Four peptides were studied, spanning the sequence of the residues 38–61 of wild-type ANXA11 and mutated versions (WT, G38R, and D40G). The peptides were purchased from PEPCEUTICALS Ltd. (Leicester, United Kingdom). The molecular weights were validated by mass spectrometry.

### ANXA11 expression and purification

The coding sequence for full-length human ANXA11 in the plasmid pMCSG7-MBP-ANXA11 was kindly sent by Boris Rogelj’s laboratory (Jožef Stefan Institute, Ljubljana, Slovenia). The ANXA11 N-terminus (residues 1–191) was donated by Salvatore Adinolfi (University of Turin, Italy) in a pET His6 TEV LIC plasmid with an N-terminal thioredoxin tag. The constructs were expressed in *E*. *coli* BL21 (DE3) cells.

Transformed cultures of the proteins were grown in Luria broth (LB) supplemented with 100 μg/mL ampicillin at 37°C until the optical density at 600 nm reached 0.6 and induced with 1 mM isopropyl β-d-1-thiogalactopyranoside (IPTG) at 20°C. Cells were collected by centrifugation and resuspended in lysis buffer and lysed by sonication. The soluble proteins were recovered in the supernatant by centrifugation at 4°C, and purified by nickel affinity chromatography using 300 mM imidazole in the elution buffer. The tag of ANXA11 was cleaved by incubating the construct with tobacco etch virus protease (1:50 enzyme/protein) overnight at 4°C, while dialyzing the mixture in SEC buffer. Pure ANXA11 was obtained after a reverse Ni-NTA chromatography step, and a further size-exclusion chromatography step on an Äkta pure system (HiLoad 16/600 Superdex 200 column, GE Healthcare). Each purification step of the full-length protein was carried out at 4°C, at pH 8.5 in 20 mM Tris-HCl buffer, 150 mM KCl, and additional 5 mM EDTA and 1 mM DTT in the SEC buffer.

Pure ANXA11 N-terminus with an N-terminal Thioredoxin-(His)6 tag was obtained after a further step of size-exclusion chromatography on an ÄKTA Prime Plus system (HiLoad 26/600 Superdex 75 prep grade column, GE Healthcare). Attempts to purify the untagged ANXA11 N-terminus in 20 mM HEPES, 20 mM NaCl buffer at pH 7.0 without the Thioredoxin-(His)6 tag led to quantitative precipitation. Protein purity was assessed by SDS-PAGE (Supplementary Figure S2). Protein identity was validated by LC-MS/MS with 86% of sequence coverage. The proteins were aliquoted, flash-frozen and stored at −80°C. ^15^N-labelled samples were obtained by growing the cells in minimal media using ^15^N ammonium sulphate as the sole source of ammonium.

### Spectroscopic measurements

Far-Ultraviolet (UV) CD spectra were recorded on a JASCO-1100 spectropolarimeter equipped with a temperature control system, averaged over 10 scans and deconvoluted with the online analysis software K2D3 and BestSel. Measurements were carried out in 1 mm path-length quartz cuvettes (type S3/Q/1; Starna Scientific), applying a constant N_2_ flush at 4.0 L/min.

NMR measurements were carried out on 200 µM non-labelled peptides in a 10 mM sodium phosphate buffer, at pH 6.8 and on 100 µM ^15^N-labelled Thioredoxin-tagged ANXA11 N-terminus sample in 20 mM HEPES, 20 mM NaCl buffer at pH 7.0. D_2_O (10%) was added to the samples. NMR spectra were recorded on Bruker 800 spectrometers at 5°C and at 25°C, respectively, and processed with NMRPipe (Delaglio et al., 1995). Spectra were analyzed and assigned with NMR-Fam Sparky (Lee et al., 2015) and CARA 1.9.1.7 (Keller, 2004). TOCSY spectra were measured using the ‘dipsi2esfbgpph’ pulse sequence. NOESY spectra were recorded with a mixing time of 250 ms. Both experiments and COSY spectra (cosydfesgpphpp) were recorded with 4,096 data points in t2 and 1,024 data points in t1. For the assignment of the Thioredoxin tag a set of three spectra were recorded: ^1^H-^15^N HSQC (hsqcfpf3gpphwg_f1180), TOCSY-HSQC (dipsihsqcf3gpwg3d) and NOESY-HSQC (noesyhsqcf3gpwg3d_cpds).

### Molecular dynamics simulations

The standard iterative protocol was used with the ARIAweb 07d7d10a (2021-06-08) service (Brünger et al., 1998; Allain et al., 2020) on the Pasteur@Galaxy cluster (doi 10.7490/f1000research.1114334.1) using default settings. ARIAweb implements the latest release of ARIA version 2.3 (Rieping et al., 2007) in combination with CNS version 1.21 (Brunger, 2007) modified with dedicated ARIA routines. 143 NOE based distance restraints were used (75 intra-residue and 68 sequential). The number of structures calculated was twenty for iterations 0–8 of which the seven best - based on the total energy - were used in the proceeding iteration. After nine iterations were completed, the 10 lowest energy conformers were refined in a shell of water molecules.

Further Molecular dynamics (MD) simulations were performed using the NAMD 2.13 package (Phillips et al., 2020) with the CHARMM36m force field. Input files were generated with CHARMM-GUI (Jo et al., 2008; Lee et al., 2016). The structures were solvated with the TIP3P water model in a rectangular box such that the minimum distance to the edge of the box was 10 Å under periodic boundary conditions. An appropriate number of Na^+^ counterions were added to neutralize the protein charge. The replicas were used for three separate production runs: ii) one imposing all NOE restraints, ii) one imposing only NH-NH NOE restraints, and iii) one with no restraints. For all active restraints the lower and upper walls were defined below and above 2.5 and 5.5 Å, with constants of 2 and 10 kcal mol^−1^ Å^−2^ respectively. Each replica was subject to 1 ns of equilibration at 278.1 K and normal pressure. The production runs (100 ns) were performed under the same conditions.

The structure evaluation and analysis procedures were performed with NMRBox (Maciejewski et al., 2017). Ten thousand structures were generated.

### Computer-assisted predictions

Structure predictions were carried out either running AlphaFold 2.2.0 in NMRBox (https://nmrbox.nmrhub.org/) or running the prediction on the Baker’s lab Robetta server (https://robetta.bakerlab.org/results.php?id=550799) using the full-length protein. Per-residue estimates of the confidence of the models for the AlphaFold models are given by per-residue predicted local distance difference test (pLDDT) scores of the final model. This score is a scale 0–100 and represents the confidence of the predicted structure compared to the “true” (ground truth) structure. Likewise, confidence in RosETTAFold models is given by the global distance test (GDT) (100.0 good, 0.0 bad). The structures were visualized using Pymol (Schrödinger, L. & DeLano, W. (2020), retrieved from http://www.pymol.org/pymol).

Structure predictions of the peptides based on chemical shift information was achieved by CS-Rosetta (https://csrosetta.chemistry.ucsc.edu/) (Shen et al., 2008). CS-Rosetta produces reliable structural ensembles from NMR observables (chemical shifts, J-couplings, NOEs, residual dipolar couplings, etc.). To do this, it performs a selection of protein backbone fragments from high-resolution structures from the PDB, which are used in conjunction with Rosetta’s high-resolution energy function to model the structures of proteins up to 35 kDa.

The *cat*GRANULE software (http://service.tartaglialab.com/new_submission/catGRANULE) was used to predict the protein tendency to phase separate (Bolognesi et al., 2016). *cat*RAPID *signature* was used to predict the propensity of TDP-43 to interact with RNA and identify RNA-binding domains (Livi et al., 2016).

### SAXS measurements

SAXS experiments were performed at the BM29 beamline at the ESRF in Grenoble, France (Tully et al., 2023). The wavelength of the beamline was 0.99 Å (12.5 KeV), with the distance between sample and detector (PILATUS3 2M) set to 2,812 mm, giving the scattering vector *q* 0.007–0.55 Å^−1^. This vector is defined as *q* = 4*π* sin(*θ*)/*λ*, where 2*θ* is the scattering angle and *λ* is the wavelength of the incident beam. Ten successive frames of the scattering from the samples were recorded in batch mode with an exposure time of 2 s for each frame due to 75 mA beam intensity. The scattering from the corresponding buffer was measured before and after each sample for the same exposure time, and subtracted from the sample scattering. Measurements were performed at 20°C, and the forward scattering, *I*_0_, was converted to an absolute scale by water calibration. The data were automatically reduced using FreeSAS (Kieffer et al., 2022) and further processed and analyzed using the Scatter IV (Tully et al., 2021) and ATSAS program packages (Manalastas-Cantos et al., 2021). *I*_0_, *D*_max_ and *R*_g_ were determined from *P*(*r*), although the Guinier approach was also used for comparison. The molecular weight of the species in solution was determined from *I*_0_.

Solutions of full-length ANXA11 were measured at protein concentrations of 3.8, 3.0, 1.9 mg/mL (70.0, 55.3, 35.0 µM) in the absence of calcium and at 3.6, 2.6 and 1.8 mg/mL (66.4, 47.9, 33.2 µM) in the presence of calcium (500 µM). Interparticle interactions were seen at higher concentrations. The curves were extrapolated to 0 and scale-merged using the PRIMUS software. *Ab initio* models were constructed using the programs DAMMIN (Svergun, 1999), DAMMIF (Franke and Svergun, 2009) and GASBOR (Svergun et al., 2001). DAMMIN and its reimplementation DAMMIF represent the protein molecule by compact beads connected to each other. In GASBOR, proteins are represented as an ensemble of dummy residues instead of dummy atoms. The crystal structure of rat ANXA11 (6tu2) as a monomer was fitted manually into the GASBOR bead-model to observe the volume occupied by the N- and C-terminus. Molecular ensemble models were generated by the Ensemble Optimization Method (EOM) software (Bernado et al., 2007; Tria et al., 2015). This package generates an ensemble of protein conformations and a theoretical average scattering intensity curve based on the ensemble. Finally, it fits the theoretical curve onto the experimental SAXS data. High-resolution structures of individual protein domains can be used as rigid bodies, while intrinsically disordered protein segments are modelled with completely random configurations. The RANCH, FFMAKER and GAJOE programs, all available at https://www.embl-hamburg.de/biosaxs/, allow respectively generation of an ensemble of models, computation of the scattering intensities based on PDB structures, and the selection of the ensemble of conformations whose computed scattering curve best-fits the experimental SAXS curve. We separated the sequence of ANXA11 to an N-terminal (chain B) disordered region and a C-terminal globular domain (chain A). The C-terminal part was fixed as rigid body using the PDB file 6tu2 as a monomer. The last residue of the N terminus was kept in steric proximity of the first residue of the C-terminus by defining a 5–7 Å distance constraint between them. The input scattering curves were extrapolated to 0 concentration in the absence and presence of calcium. The raw data were deposited to the SASBDB database with accession codes SASDTV5 (apo) and SASDTW5 (holo).

## Results

### The N-terminal domain is intrinsically disordered

**FIGURE 1:**
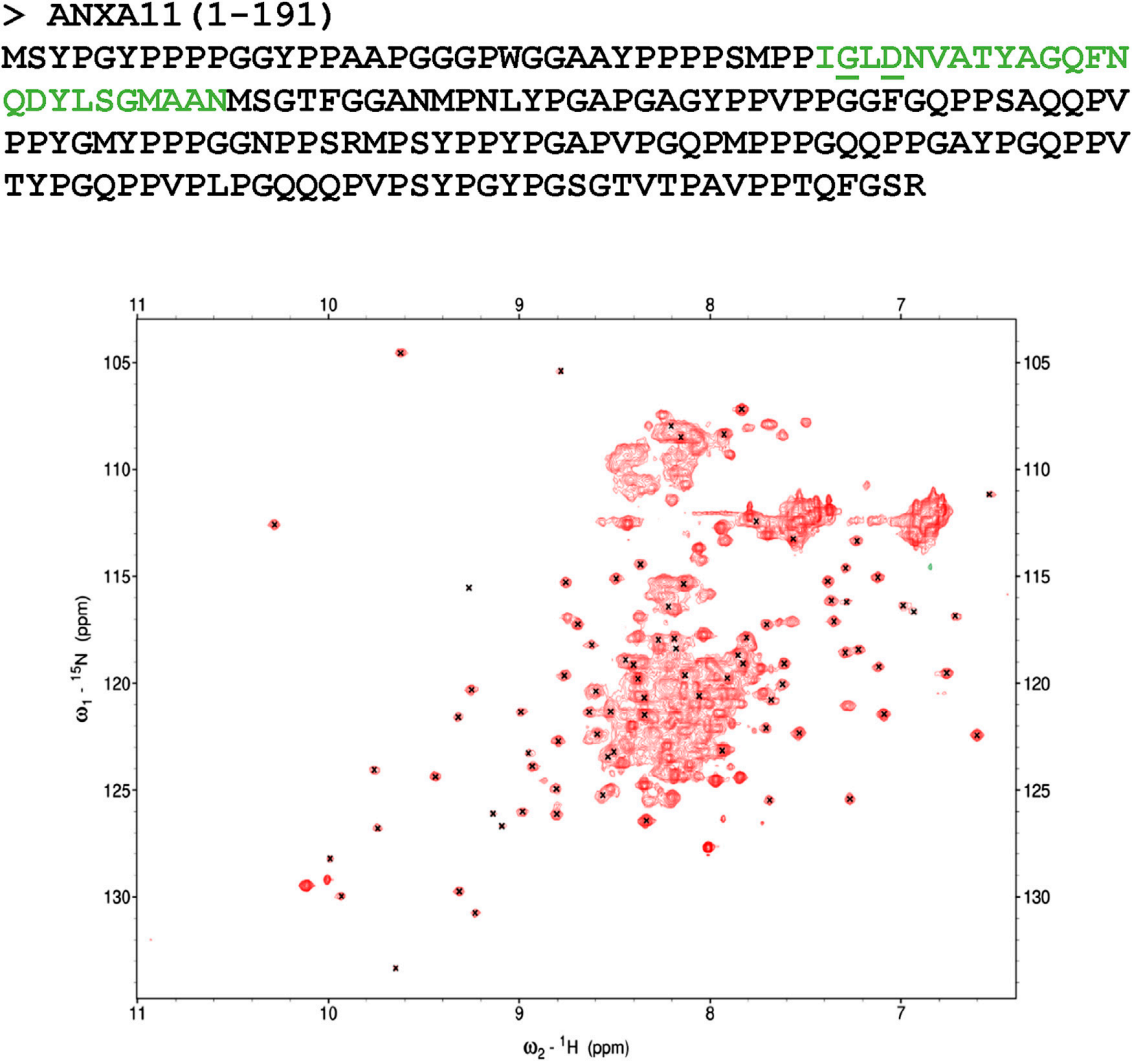
Characterization of the N-terminus of ANXA11. Top panel: amino acid sequence of the N-terminus of ANXA11 (residues 1–191). The position of the putative helix is indicated in green. The clinically important residues 38 and 40 are underlined. Bottom panel: ^1^H-^15^N HSQC spectrum of the thioredoxin-His-tagged ANXA11 N-terminus at 298 K and 800 MHz. Crosses indicate the resonances of Thioredoxin according to the BMRB assignment. Most of the spread of the spectrum thus correspond to the Thioredoxin contributions.

We produced the recombinant N-terminal domain of ANXA11 by *E. coli* expression of a fusion protein with an N-terminal thioredoxin tag. Since attempts to cleave the tag led to quantitative precipitation of the protein, we decided to keep the tag and analyze the fusion protein by NMR. The spectrum of this ^15^N-labelled protein showed a good dispersion but with a large number of resonances overlapping in the center of the spectrum (Figure 1). Comparison of the spectrum with the spectral assignment of thioredoxin retrieved from the BMRB data base (27636) allowed us to establish that the vast majority of the resonances with good chemical shift spreading correspond to residues in the thioredoxin tag, indicating that the residues of the ANXA11 N-terminus mainly contribute to the spectrum by the overlapping resonances. Absence of appreciable shifts of the thioredoxin peaks from their positions in the isolated protein indicated lack of significant interactions between the two proteins. This observation tells us that the ANXA11 N-terminus is mostly unstructured, as expected from the sequence composition.

### Synthetic peptides have intermediate features between random coil and helical structures

**FIGURE 2:**
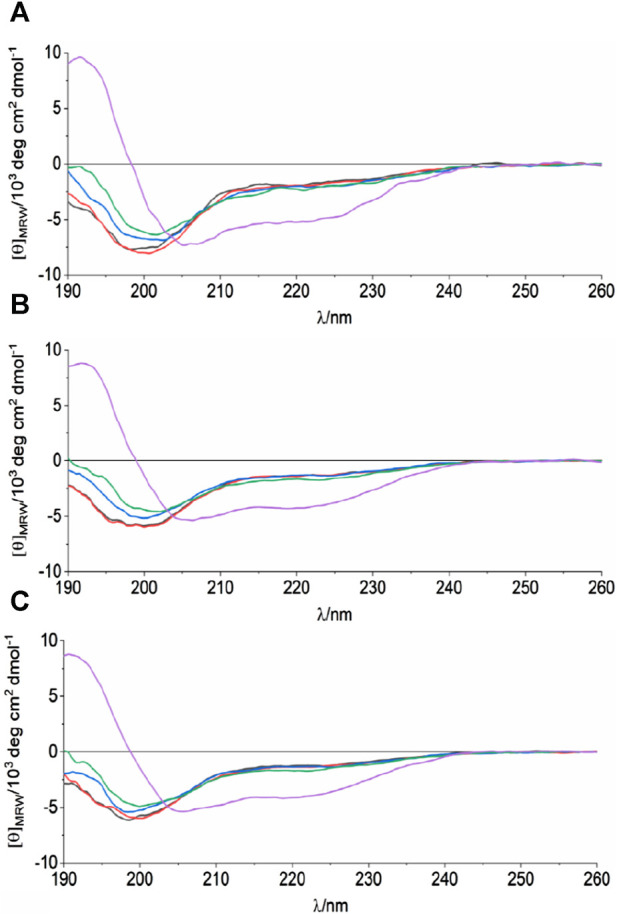
CD analysis of the synthetic peptides spanning residues 38–59 of ANXA11. The spectra correspond to WT **(A)**, G38R **(B)** and D40G **(C)** mutant peptides at 298 K, in 10 mM sodium phosphate, pH 6.8 and different TFE concentrations: 0%—black, 1%—red, 5%—blue, 10%—green, 30%—purple.

We then analyzed the structure of the region 38–59 using synthetic peptides: we used a peptide spanning the sequence of wild-type ANXA11 (hereafter referred to as WT) and three peptides in which the clinically important mutations G38R and D40G were introduced. These mutants are hereafter indicated as G38R and D40G peptides. We screened different pH, temperatures, and buffer conditions by far-UV CD to understand how they could affect the WT peptide. When the peptide was dissolved in 10 mM phosphate buffer, it gave a CD spectrum with a negative minimum in ellipticity at 200 nm (Figure 2). This behavior is typical of an unfolded conformation. However, the spectrum also had a weak negative band around 220 nm which was compatible with a residual helical structure in equilibrium with a random coil conformation in the CD time scale (native helix) (Dyson et al., 1988a). Changes in temperature and buffer/pH did not significantly affect the amount of secondary structure of the peptide. The K2D3 webserver (Louis-Jeune et al., 2012) estimated 3%–6% α-helical and 15%–17% beta strand content. When 1%–30% (v/v) trifluoroethanol (TFE) was added, the minimum at 223 nm became deeper, corresponding to an appreciable increase in the propensity to a helical secondary structure. This alcohol is known to stabilize helical structure in peptides and is often used to enhance their helical propensities (Vincenzi et al., 2019). The G38R and D40G mutant peptides did not show appreciable differences to the WT. These results support the hypothesis of a helical element in this region of the ANXA11 N-terminus.

### The 38–59 region has a strong helical tendency as observed by NMR

**FIGURE 3:**
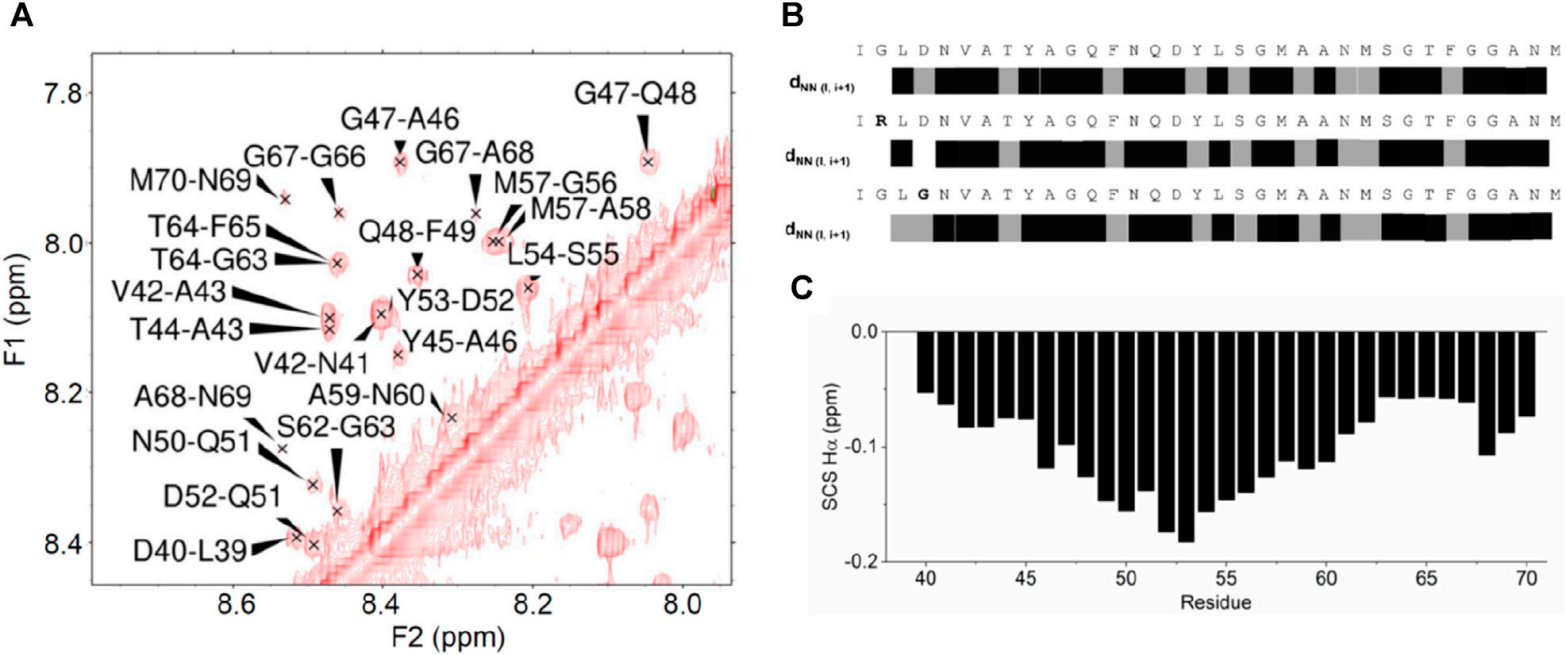
NOE effects observed for the ANXA11 peptides. **(A)** Amide region of a 2D NOESY spectrum of the wild-type peptide. **(B)** Diagrammatic representation of the sequential NOE HN-HN connectivities along the ANXA11 peptides. Connectivities marked in gray indicate that the sequential amide-amide NOE peak was close to the diagonal or had low intensity and thus was ambiguous. **(C)** Plot of the secondary chemical shifts, that is the difference between the observed chemical shifts and the random coil values of the same residue, along the sequence, averaged according to Pastore and Saudek (1990).

CD spectroscopy is an excellent technique to screen conditions, but NMR is a much more powerful means as it works in a different average time scale and can provide sequence-specific information on the structure of peptides. We thus studied the structural behavior of the peptides by NMR. Virtually complete assignment of the NMR spectrum of the non-labelled WT peptide in aqueous buffer was obtained using standard 2D techniques (Wuthrich, 1986; Redfield, 1993), except for the highly mobile and solvent exchangeable first and second N-terminal residues. Numerous Nuclear Overhauser Effects (NOEs) were observed that are typical of an α-helical conformation. This is unusual for a peptide of this relatively small size in aqueous solutions, even more at neutral pH and without the addition of co-solvents. In particular, an almost uninterrupted network of sequential HN-HN effects was observed along the whole sequence (region L39-N69) (Figure 3). This behavior indicated a strong tendency of the peptide to fold in a helical conformation throughout the sequence and confirmed an overall behavior typical of a nascent helix (Dyson et al., 1988b). The 10 lowest energy models of the WT peptide obtained by CS-Rosetta, a structure prediction program that uses chemical shift information, contained flexible termini (residues 37–38, 66–69), two distinct alpha-helices (residues 39–47, 52–65) and a short turn connecting the helices. This prediction does not however reflect the uninterrupted NH-NH sequential NOE cross-peak pattern. Accordingly, no long-range NOEs characteristic for a hairpin-like structure was identified. A plot of the secondary chemical shifts of the Hα of the peptides as defined by Pastore and Saudek (1990), a simple but effective method to detect secondary structural tendencies, suggested an uninterrupted helical structure for the isolated peptide. Conversely, extensive restrained and unrestrained molecular dynamics simulations provided trajectories with only transient formation of local helical regions, in agreement with the transient nature of a native helix (Supplementary Figure S3). Taken together, these results indicate that the peptides have a uniform tendency along the sequence to fold as a helix, but this secondary structure is not stably formed in water in the absence of stabilizing tertiary contacts.

When we analyzed the mutants, only minor chemical shift differences were observed at and around the residues affected by the mutations. Accordingly, the overall NOE patterns remained unchanged, indicating that the mutations do not affect the peptide structure and thus suggesting that D40 has a functional role. This is in agreement with the observation that the D40G mutation abolishes calcyclin binding (Smith et al., 2017).

### Structural predictions of the full-length protein

**FIGURE 4:**
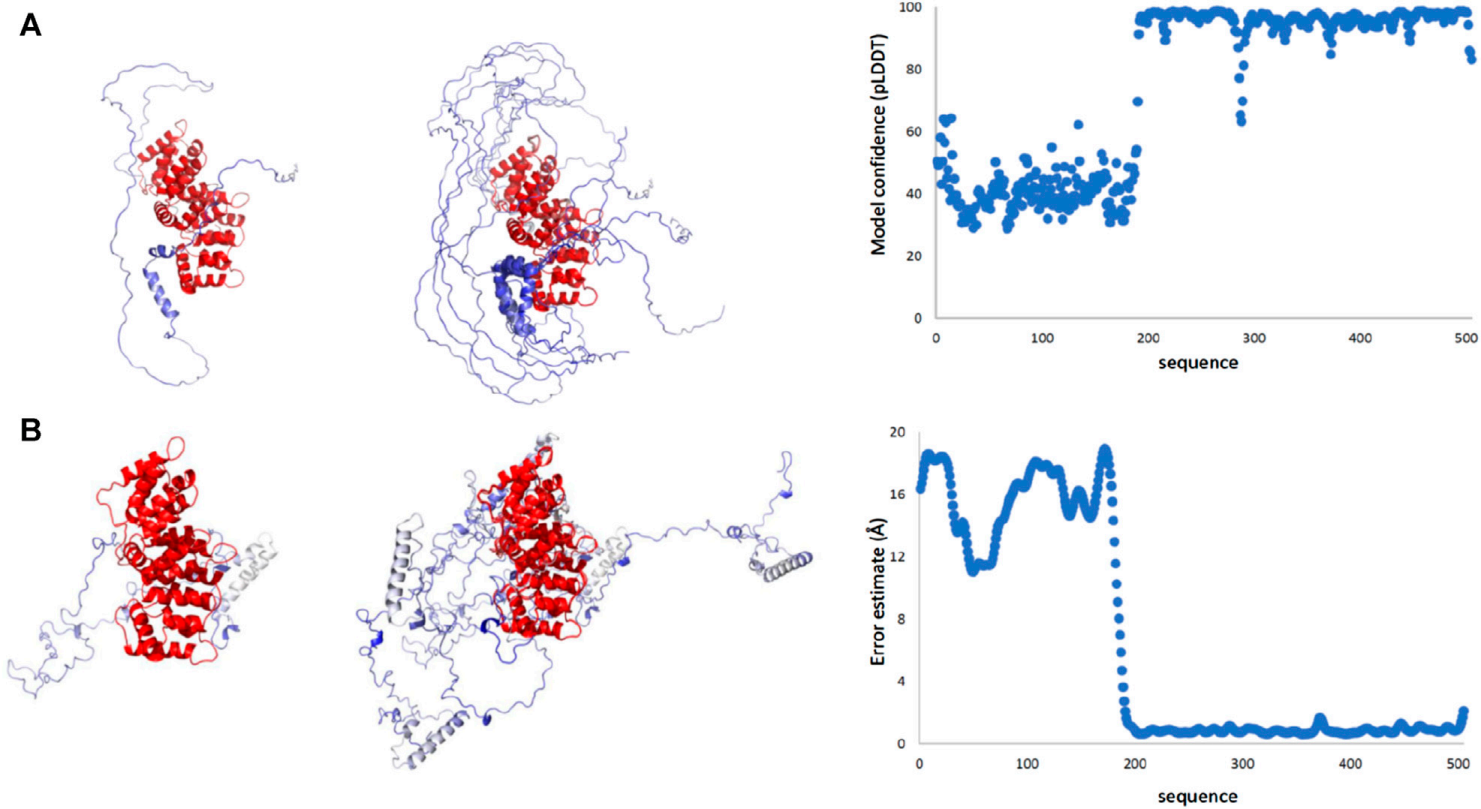
Structural predictions of ANXA11 by RosETTAFold and AlphaFold. **(A)** The best-score prediction from AlphaFold (left), a bundle of the best-score predictions (middle), and a plot of the score confidence (pLDDT) *versus* the amino acid sequence (right). Values of pLDDT >90 indicate a model with high accuracy; values 70 to 90 a generally good backbone prediction; 50 to 70 low confidence, and <50 not reliable. **(B)** The best-score prediction from RosETTAFold (left), bundle of the best-score predictions (middle), and error estimate (right). The two plots provide a different but complementary version of the prediction reliability. In both sets of models, the reliability of the C-terminus is high, reflecting the level of conservation, whereas the N-terminal domain has much lower reliability.

To gain more information on the full-length protein, we first consulted AlphaFold (Jumper et al., 2021; Varadi et al., 2022) and RoseTTAFold predictions (Yang et al., 2020). Both predictions detect the presence of two distinct domains. The reliability of the C-terminus is high, which reflects high pLDDT values, whereas the non-conserved N-terminal domain has low confidence (Figure 4A). This is reasonable since, while the multiple alignment of the C-terminus comprises sequences and structures from all members of the vast annexin family, the N-terminus is specific to ANXA11 and the domain is intrinsically disordered as shown above by NMR.

The predictions by the two servers are different but have one feature in common (Figure 4B): all models predict an overall disordered structure for the N-terminus with a helix around residues 38–59. In the Alphafold structures, the helix tends to be interrupted around residues 47–49, whereas in the RosETTAFold structures the helix is uninterrupted. In the AlphaFold structures, the N-terminus consistently wraps around the C-terminal domain creating a more globular, though expanded, structure with the possibility of making contacts also between the region 38–59 and the first two annexin repeats. In the RosETTAFold structures, the N-terminus is completely separated from the C-terminus and does not form interactions with it.

### SAXS suggests a conformational ensemble dominated by more globular species

SAXS is a low-resolution structural technique which can provide information about overall shape and domain orientation of proteins and is well suited to investigating flexible or intrinsically disordered proteins (Kachala et al., 2015; Lenton et al., 2023). We characterized full-length ANXA11 both in the presence (holo) and in the absence (apo) of a 5 M excess of CaCl_2_. The excluded volumes of the hydrated particles (V_p_) for the holo and apo ANXA11 were reasonably consistent with the values expected for a monomeric species and with the masses estimated from the primary sequence (Table 1). The higher than expected, estimated values of the molecular weight, ∼74–62 kDa, as compared to the theoretical one (54.4 kDa) are likely explainable by the flexibility of the system and to a minor degree of aggregation that appeared to be more accentuated in the calcium-free samples, as it is evident from some concentration dependence of the SAXS observables in the experiments in the absence of calcium.

**TABLE 1:** Summary of the SAXS paramaters.

Data-collection parameters	
Instrument	ESRF BM29
Wavelength (Å)	0.99
q-range (Å^−1^)	0.007–0.5
Sample-to-detector distance (m)	2.8
Concentration range (mg/mL)	1–4
Temperature (K)	293
Detector	Pilatus P3-2M
Flux (photons/s)	1^a^10^13^
Beam size (µm)	500^a^200

Structural parameters (absence of Ca^2+^)	3.8 mg/mL	3.0 mg/mL	1.9 mg/mL	0 mg/mL, extrapolated
I_0_ (kDa) [from Guinier]	2.4	1.7	0.9	1.9
I_0_ (kDa) [from real space]	2.3	1.6	0.9	1.9
R_g_ (Å) [from Guinier]	42.5 ± 0.7	40.8 ± 0.7	37.3 ± 0.7	35.5 ± 0.7
R_g_ (Å) [from real space]^a^	44.8	41.5	38.7	38.4
qmin—qmax used for Guinier (Å^−1^)	1.2 × 10^−2^–3.1 × 10^−2^	8.3 × 10^−3^–3.2 × 10^−2^	8.9 × 10^−3^–3.5 × 10^−2^	6.8 × 10^−3^–3.7 × 10^−2^
Volume (Å^3^) real space	1.1 × 10^5^	1.0 × 10^5^	9.9 × 10^4^	9.8 × 10^4^
D_max_ (Å)^a^	172.5	166.0	142.0	154.5
Calculated MW (kDa)	74	67	64	63
Calculated theoretical MW monomer (kDa)	54	54	54	54

Structural parameters (presence of Ca^2+^)	3.6 mg/mL	2.6 mg/mL	1.8 mg/mL	0 mg/mL, extrapolated
I_0_ (kDa) [from Guinier]	2.4	1.5	0.9	2.0
I_0_ (kDa) [from real space]	2.1	1.4	0.8	1.8
R_g_ (Å) [from Guinier]	43.6 ± 1.0	40 ± 0.8	38.94 ± 0.6	37.52 ± 0.6
R_g_ (Å) [from real space]^a^	43.6	41.5	38.1	38.0
qmin—qmax used for Guinier (Å^-1^)	1.1 × 10^−2^–3.0 × 10^−2^	1.3 × 10^−2^–3.2 × 10^−2^	6.8 × 10^−3^–3.4 × 10^−2^	7.3 × 10^−3^–3.5 × 10^−2^
Volume (Å^3^) real space	1.0 × 10^5^	1.0 × 10^5^	9.4 × 10^4^	9.5 × 10^4^
D_max_ (Å)^a^	172.0	166.0	140.0	150.0
Calculated MW (kDa)	66	65	61	61
Calculated theoretical MW monomer (kDa)	54	54	54	54

Software employed	
Primary data reduction	BM29 autoprocessing pipeline
Data processing	Scatter IV, PRIMUS, DAMMIN, DAMMIF, GASBOR, EOM

^a^ Uncertainties are not given by the program Scatter IV.

**FIGURE 5:**
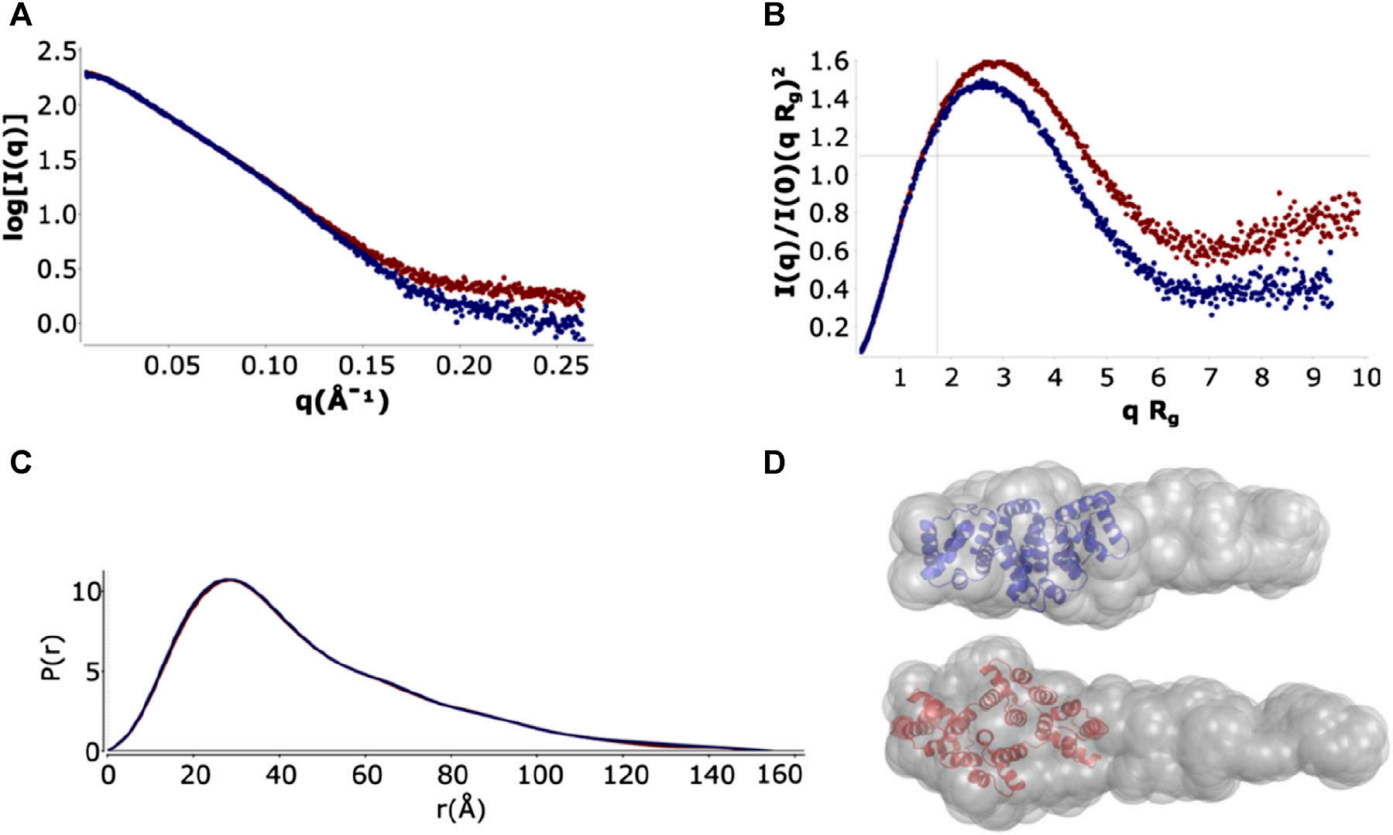
SAXS analysis and *ab initio* model of full-length ANXA11 in the presence and absence of calcium. **(A)** Plot of the Log_10_ SAXS intensity *versus* scattering vector, *q*. *Dark blue*: ANXA11 in the absence of calcium. *Dark red*: ANXAA11 in the presence of 500 µM calcium extrapolated to 0 mg/mL protein concentration. **(B)** Dimensionless Kratky plot. Cross-hair marks the Guinier-Kratky point (1.7, 1.1), the main peak position for globular particles. **(C)** Pair-distance function, P(r). The maximum dimension, *D*_*max*_, is the largest non-negative value that supports a smooth distribution function. **(D)** GASBOR *ab initio* models from scattering curves extrapolated to 0 concentration.

The log I(q) *versus* q curves showed a high degree of similarity at low q, with the holo form having a slightly larger R_g_. A small deviation was seen at high q (Figure 5A and Supplementary Figure S4) that is consistent with small changes in buffer matching. The main peak of the normalized Kratky plots has an approximately gaussian shape that indicates a globular, elongated domain, whereas the tail suggests an unfolded region (Figure 5B), as explained in more detail below. Pair-distribution curves for the holo and apo forms showed a similar bell-shape with elongated tails with an increased D_max_ 154.5 ± 0.5 Å for the apo form as compared with 150 ± 0.5 Å with the holo form. The computed distance distribution functions P(r) displayed a single peak with a tail, a pattern that is typical of proteins with elongated shapes (Figure 5C).

Shape reconstruction of full-length ANXA11 was performed by *ab initio* modelling using two complementary programs, DAMMIN (Svergun, 1999) and GASBOR (Svergun et al., 2001). The crystal structure of the C-terminus (6tu2) fitted well into one end of the more detailed GASBOR model leaving a region of extra density for one possible conformation in the ensemble of flexible N-terminus intrinsically disordered region to fill (Figure 5D).

**FIGURE 6:**
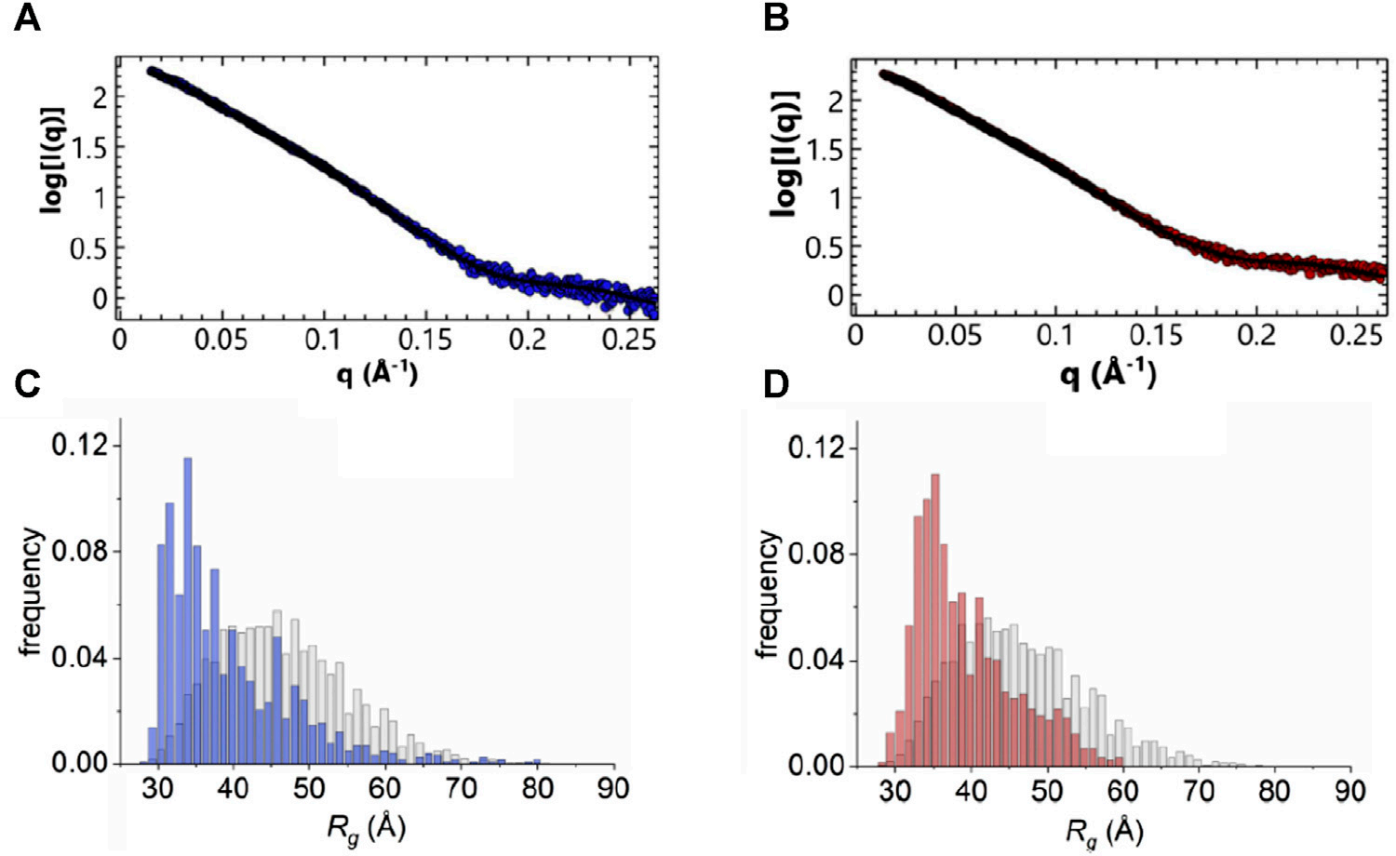
EOM analysis. **(A, B)** Fitting of an averaged theoretical scattering intensity derived from an ensemble of conformations using EOM (black) to experimental SAXS data extrapolated to 0 mg/mL protein concentration (blue: in the absence of calcium, red: 500 µM in the presence of calcium). **(C, D)** Plot of R_g_ distributions from EOM analysis, pool frequency (grey) and selection frequency (blue and red, absence and presence of calcium, respectively).

The flexibility of the N-terminus was further investigated using two different approaches. For a qualitative approach, we used the normalized Kratky plot (Figure 5B) (Durand et al., 2010), that allows direct comparison of objects with different shapes and sizes. In this plot, folded compact globular proteins provide a bell-shaped curve at low angles with a maximum at q*R_g_ of 1.75 (Durand et al., 2010). We observed instead Kratky plots with maxima at q*R_g_ of 2.6 and 2.8 for the apo and holo ANXA11. These deviations from the standard behaviour are consistent with an appreciable level of flexibility. Both plots showed a broadening of the bell-shaped curve and a shift of the maxima to larger q*R_g_ values, as expected for extended and flexible molecules. The plots were also characterized by upward trends at higher q*R_g_ values (i.e., higher scattering angles), which is also indicative of flexibility. To obtain a more quantitative approach, the ensemble optimization method (EOM) (Bernado et al., 2007) was used to analyze the flexibility and size distribution of possible multiple configurations and to obtain optimized ensembles with a fit to the experimental scattering data (χ^2^ ∼1.68 and 1.95) (Figure 6). The values of the ensemble average R_g_ from the EOM analysis were 39.7 and 39.8 Å in the absence and in the presence of calcium. Likewise, no significant variation was observed between the ensemble average D_max_ values, 148.4 and 149.2 Å. The values showed reasonable agreement with the experimental data. The degree of flexibility of apo and holo ANXA11 was estimated by comparing the corresponding Shannon entropy R_flex_ values of the ensemble distributions to that of a random pool and is a reference for flexibility. The comparison between apo and holo ANXA11 revealed almost identical ensemble R_flex_ values (77% *versus* 74%, respectively) and confirmed random motions of the N-termini. Looking in greater detail to the R_g_ distributions of these ensembles, we see the ensembles were shifted to the left of the distribution of randomly generated models of the initial pool (Figure 6). This indicates that the ensemble of conformations is more often at a lower R_g_ and thus more globular rather than completely elongated, suggesting that the flexible regions may be predominately around the core than fully extended (Supplementary Figure S5). This conclusion leads us to consider the solutions from the Alphafold server more dominant within the conformational ensemble compatible with the SAXS measurements.

Altogether, these results tell us that there is little difference between the structures of the apo and holo forms of ANXA11, suggesting that calcium regulation does not involve major conformational changes between the calcium free and calcium loaded forms. This is in agreement with what is observed in other members of the annexin family with a much shorter N-terminus (Burger et al., 1996; Sopkova et al., 2002; Rosengarth and Luecke, 2003; Hong et al., 2020). It also tells us that the N-terminus does not appreciably participate to the calcium regulation of ANXA11 which seems to involve mainly the C-terminus.

### Functional peculiarities of the N- and C-termini of ANXA11

Finally, we analyzed the question of which region(s) of ANXA11 is/are involved in soluble (liquid-to-liquid) phase separation and in RNA-binding. We assessed the potential of ANXA11 to form protein granules using the catGRANULE approach (Bolognesi et al., 2016). catGRANULE is a machine learning software trained on granule-forming proteins, utilizing features such as RNA binding, structural disorder, and amino acid composition to predict phase separation propensity. It specifically considers factors such as structural disorder, nucleic acid binding affinity, and amino acid motifs like arginine-glycine and phenylalanine-glycine as indicative of a protein’s tendency to coalesce into granules (Bolognesi et al., 2016).

**FIGURE 7:**
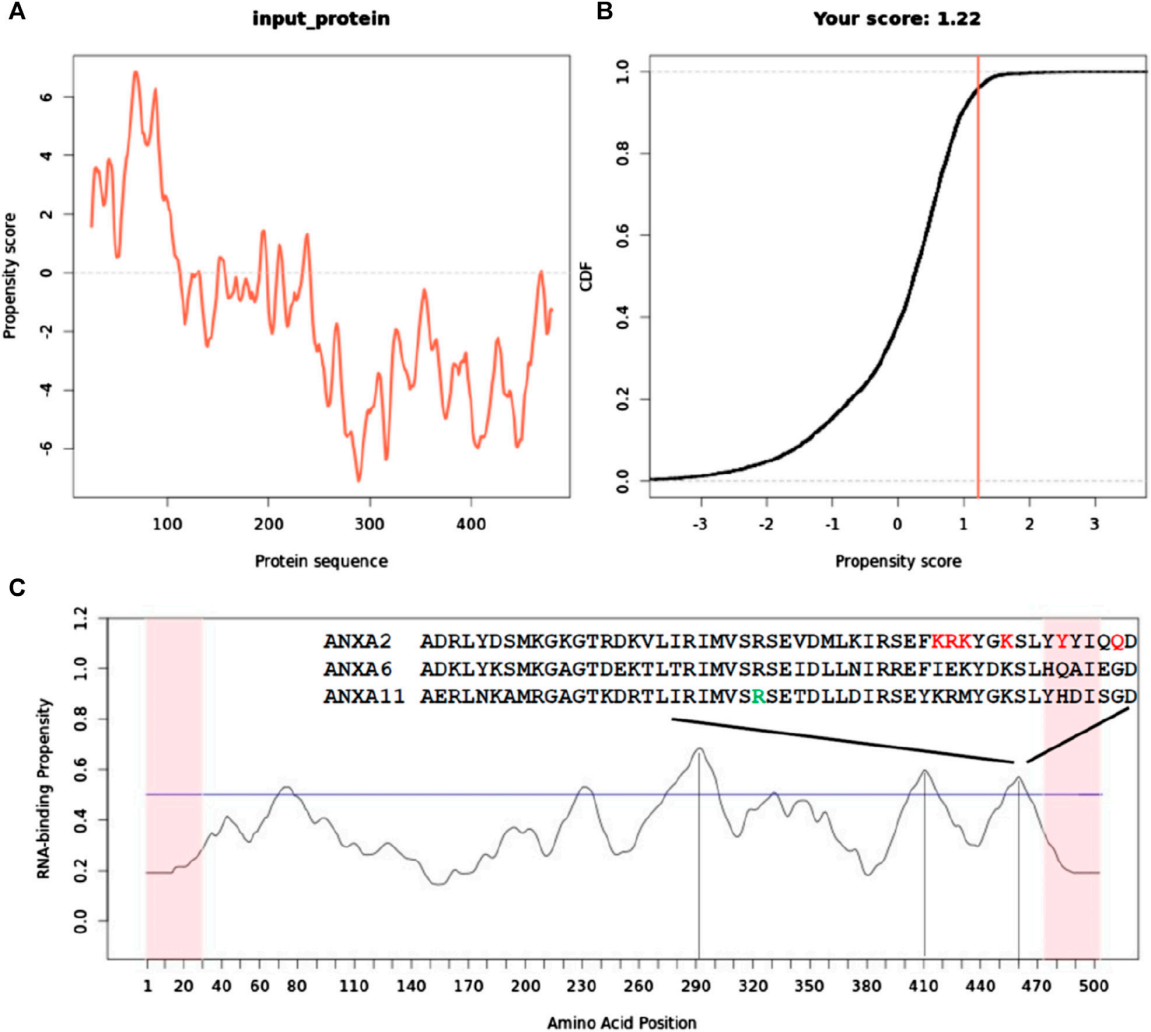
Sequence-specific prediction of the tendency of ANXA11 to promote liquid-liquid phase separation and bind to RNA. **(A)** catGranule profile of the tendency to have phase transitions having a basic minimum threshold of 0.0 for phase transition propensity. **(B)** Plot of cumulative distribution function (CDF) as a function of the propensity score. CDF describes the probability of a random variable having values less than or equal to x. It is a cumulative function that sums together the total likelihood of an event up to that point. Its output ranges between 0 and 1. **(C)** catRAPID profile that predicts the tendence of full-length ANXA11 to bind RNA along its sequence. A horizontal line indicates the threshold for RNA binding. Note that each position on the *x*-axis corresponds to +/− 25 amino acids. Vertical lines help locating the positions along the sequences of the three higher peak maxima. The last maximum above the threshold contains a sequence semi-conserved in other annexins (indicated in the onset) that has been proven to be involved in RNA binding (Aukrust et al., 2007). The amino acid indicated in green in the alignment corresponds to residue 460 that is the local maximum in this region. Residues marked in red correspond to positions that have been shown to affect RNA binding when mutated in ANXA11 from rat (Aukrust et al., 2007).

The catGRANULE analysis indicated a strong propensity for granule formation, predominantly localized within the first ∼200 N-terminal residues, with limited contribution from the remainder of the protein (Figures 7A, B). This finding aligns with recent research proposing that the N-terminus is both necessary and sufficient for driving concentration-dependent ANXA11 phase transitions from dispersion to condensation (Nixon-Abell et al., 2023). Notably, the region with the highest phase separation propensity contains a proline-rich domain (MSGTFGGANMPNLYPGAPGAGYPPVPPGGF). This is noteworthy as proline-rich domains have been implicated in phase separation (Zhang et al., 2020) and RNA binding (Wang et al., 2006) as observed in the case of Tau.

To complement these findings, we conducted an analysis with the catRAPID signature program, which predicts RNA-binding regions within a protein sequence. catRAPID signature leverages physicochemical properties, secondary structure characteristics, and hydrophobicity profiles (Livi et al., 2016). The analysis identified three regions in the C-terminal core domain with the potential for RNA binding, while suggesting minimal RNA-binding capability at the N-terminus (Figure 7C). The absolute maximum is around residue 290 that is in the second annexin repeat. The other two lower maxima are in repeats 3 and 4. Notably, the last maximum encompassing residues around position 460 contains a sequence homologous to the consensus motif (F/Y)XXX (F/Y)XKSL, known to interact with nucleic acids/RNA in ANXA2 and ANXA6 (Bandorowicz-Pikula et al., 2003; Aukrust et al., 2007). Two smaller maxima were observed, one of which is in the N-terminus. They are however very close to the threshold and do not support a strong tendency to stoichiometric interactions. The one in the N-terminus is detected only in the context of the full-length protein.

These results point towards a primary role of ANXA11 N-terminus in granule formation, primarily triggered by structural disorder rather than RNA binding.

## Discussion

We have studied the structure of ANXA11, an underexplored member of the annexin family. Discovered in the nineties (Towle and Treadwell, 1992), ANXA11 has only recently moved into the spotlights because of its potential biological role in hitch-hiking and putative involvement in the ALS pathology. ALS-associated missense mutations seem to disrupt formation of the molecular tether that connects the N-terminus to RNP granules, and the C-terminus to lysosomes (Liao et al., 2019; Lillebostad et al., 2020). As a consequence, spinal cord neurons of ALS patients with *ANXA11* mutations have abundant cytoplasmic aggregates (Smith et al., 2017). Accordingly, *in vitro* biophysical studies have shown that ANXA11 undergoes reversible phase transition into liquid droplets and hydrogels in a process that requires the N-terminal low-complexity domain (Fernandopulle et al., 2019). The specific peculiarity of the ANXA11 sequence is its unique N-terminus that contains, within its ∼190 residues, almost one-third of prolines. In the present study we have carried out different biophysical techniques on full-length ANXA11 to characterize the protein, a task not achieved before. The full-length ANXA11 is undoubtedly a difficult protein to resolve structurally, given the presence of the long potentially flexible N-terminus and its amino acid composition. It is thus not surprising that the protein does not crystallize, while it is too small for cryo-EM studies. NMR is affordable but it requires techniques tailored for proteins proline-rich as the ANXA11 N-terminus: since prolines do not have amide groups, assignment protocols and NMR pulse sequences have been designed that specifically enable sequential assignment of proline-rich segments (Kanelis et al., 2000; Hellman et al., 2014; Karjalainen et al., 2020). They are based on modified versions of a pulse scheme that correlates intra-residue ^1^Halpha, ^13^Calpha/^13^Cbeta chemical shifts with the ^15^N shift of the subsequent residue (Wang et al., 1995). Structure predictions of the N-terminus, using computation programs such as AlphaFold and RosETTAFold approaches, can only perform with low confidence, given the relatively little number of sequences and structures that could be used in the machine-learning process.

We first showed direct evidence that the N-terminus is intrinsically disordered. Our data are independently supported by an archive preprint which draws the same conclusion on the full-length protein (Nixon-Abell et al., 2023). We then turned to study the structural features of the putative helix spanning residues 38–59. Rather than attempting to assign the spectrum of full-length ANXA11, we adopted a different strategy based on the use of synthetic peptides spanning the region under question. A similar strategy has been extensively adopted also in studies of the interactions between ANXA11 and calcyclin, and other partners (Lee et al., 2008; Rintala-Dempsey et al., 2008). We found that the WT peptide has all the features typical of a nascent helix (Dyson et al., 1988a): we observed an almost uninterrupted pattern of sequential HN-HN connectivities in the NOESY spectrum of the peptide, although medium-range NOEs characteristic of a helix could not be detected, nor could a helix be observed by CD. Upon addition of small percentages of TFE, the CD spectrum of the peptide became effectively helical. When we plotted the averaged secondary chemical shifts of the α protons according to a simple but effective method (Pastore and Saudek, 1990) which provides independent probing of secondary structure, the plot indicated the presence of a potentially uninterrupted helix. According to this evidence, all predictions detect a helical signal in the region 38–59. Interestingly, no differences were observed between the WT peptide and its clinically important mutants (Smith et al., 2017), strongly suggesting that any difference observed in *in vivo* binding of this region to partners is not due to structural but functional reasons: the p.D40G mutation which has been reported to abolish binding with calcyclin is likely to cause disruption of a direct interaction between the two proteins by replacement of a negatively charged residue with a smaller non-charged glycine which will weaken or completely abolish binding.

We then resorted to SAXS, a technique that, albeit at low resolution, provides information on the general features of a protein structure, to characterize the overall shape of ANXA11. SAXS has also been successfully used to define the conformational ensembles of intrinsically disordered proteins (Cordeiro et al., 2017). We compared the results in the presence and absence of calcium, probing important parameters such as D_max_, R_g_ and flexibility. We observed differences between the two datasets but overall the protein does not undergo major conformational changes upon calcium binding. If anything, it seems that the calcium-free ANXA11 has slightly higher tendency to aggregate, a behaviour not uncommon in calcium-binding proteins (Travé et al., 1995). This means that the N-terminus does not appreciably participate to the calcium regulation, suggesting the question of what is the role of this long low complexity region that is specific for this protein within the whole annexin family.

In a comprehensive preliminary paper, it was shown that the N-terminal domain is necessary and sufficient to promote liquid-liquid phase separation of the whole molecule which incorporates RNA into granules while interacting at the same time with lysosomes in a calcium-dependent way (Nixon-Abell et al., 2023). ANXA11 should thus act as a trait-d’union between lysosomes, which are the carriers of this hitchhiker, and RNA granules (Liao et al., 2019). It was also suggested that regulation of interactions with other proteins, such as ALG-2 and calcyclin (Rintala-Dempsey et al., 2008), occurs through a Ca^2+^-induced local conformational rearrangement of the C-terminus that propagates to the N-terminus, making it proficient for interaction with its partners (Smith et al., 2017). Our data are consistent with this possibility but could also suggest a regulation distinct from that observed in ANXA1, considering that we have no evidence of an intercalation of the N-terminus into the C-terminal core domain. Also, it should be noted that, although not supported by any direct evidence or indirect suggestion, some expectation has been created that the N-terminal domain could bind itself to RNA to establish a geographical specificity in which the C-terminal core domain binds, as in all annexins, lipids and thus liposomes, whereas the N-terminus could be specialised in binding to RNA. This possibility is reasonable although the sequence of the N-terminus does not contain any motif that could favour a stoichiometric well-defined RNA-binding. We hypothesize instead that non-specific RNA binding could be achieved by recruitment of ANXA11 in the transient granule in a non-stoichiometric way through the liquid-liquid phase separation process. Indeed, a mechanism of proline promoted trapping of RNA could actually be very interesting and in line with observations that have demonstrated the importance of prolines, the only amino acid that can exist in both conformations, and prolyl isomerases in liquid-liquid phase separation (Babu et al., 2022).

While more extensive testing is required to clarify this important aspect, it seems safe to say that the N-terminal domain of ANXA11 is an excellent example of a *bona fide* intrinsically disordered domain in which disorder is essential for the formation of phase transition probably co-adjuvated by protein-protein interactions.

## Data Availability

The datasets presented in this study can be found in the online repository SASBDB with accession codes SASDTV5 and SASDTW5.
