# Supplementary material for: The structural properties of full-length annexin A11
Source: Front Mol Biosci. 2024 Mar 7;11:1347741. doi: 10.3389/fmolb.2024.1347741 (PMC10955470; doi:10.3389/fmolb.2024.1347741)

**Supplementary Materials**

**Figure S1.** Multiple alignment of human annexins. Since ANXA6 contains eight annexin repeats instead of the usual four, obtained by gene duplication, the two repeated blocks are indicated as ANXA6 and ANXA6_1. The alignment clearly shows that the C-terminal core domain is highly conserved, whereas the N-terminus is variable both in length and sequence, with ANXA7 and ANXA11 being the longer members of the family.

**Figure S2.** SDS-PAGE gel images of SEC fractions from the last protein purification step. A. SEC fractions of ^15^N-labelled thioredoxin-His-tagged ANXA11 N-terminus, 2-4 pooled together. Expected MW 33.7 kDa. B. SEC fractions of full-length ANXA11, fractions 1-5 pooled together. Expected MW 54.4 kDa.

**Figure S3**. Structure simulations of the WT peptide extracted from differently restrained and unrestrained trajectories. Red – no restraints, green – with NH-NH NOE restraints, blue – with sequential NOE restraints. Superposition was attempted on all backbone atoms. In all runs we obtained highly flexible structures adopting a dynamic, highly flexible ‘extended loop’ conformation.


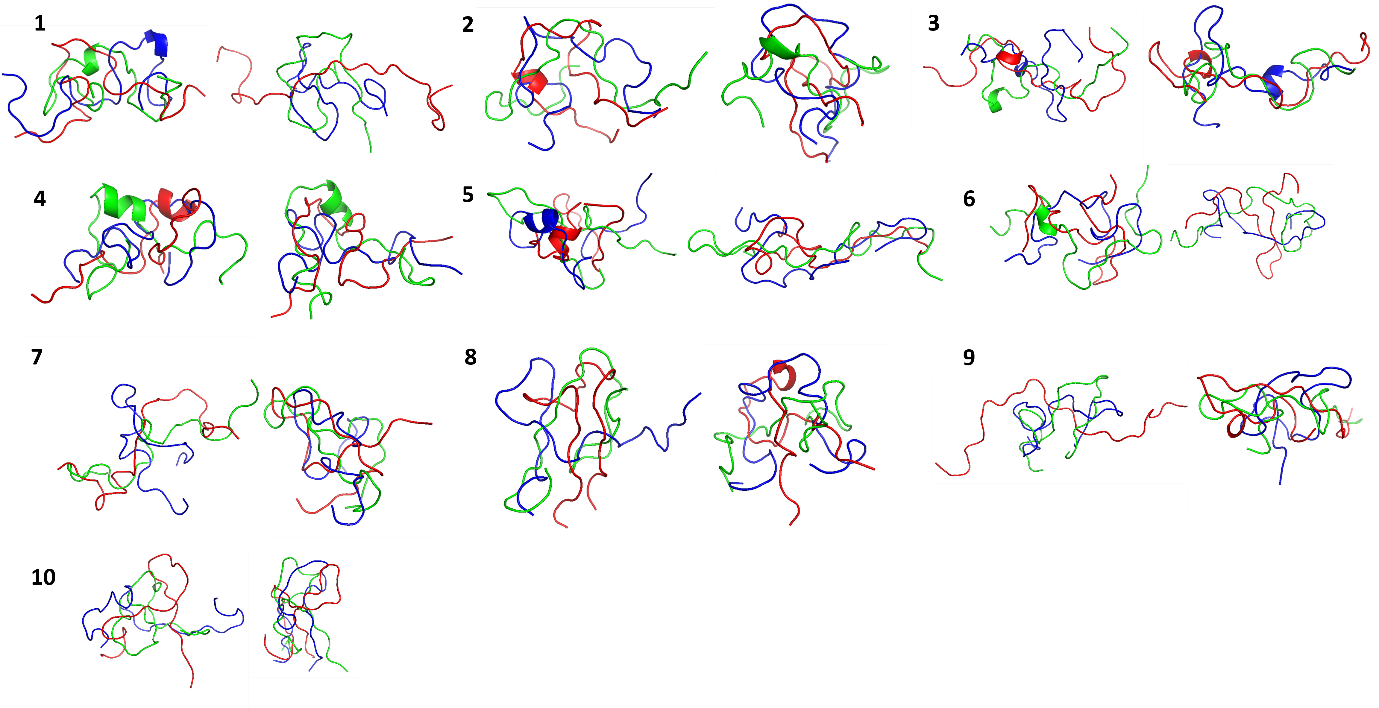


**Figure S4.** SAXS analysis of full-length ANXA11 at different concentrations. Top: Log_10_ SAXS intensity versus scattering vector, *q*. *Blue*: no calcium 3.8 mg/mL. *Middle blue*: no calcium 3.0 mg/mL. *Light blue*: no calcium 1.9 mg/mL. R*ed*: with 500 µM calcium 3.6 mg/mL. *Bright red*: with calcium 2.6 mg/mL, *pink*: with calcium, 1.8 mg/mL. Bottom: Pair-distance function, P(r). Maximum dimension, *d_max_*, is the largest non-negative value that supports a smooth distribution function.


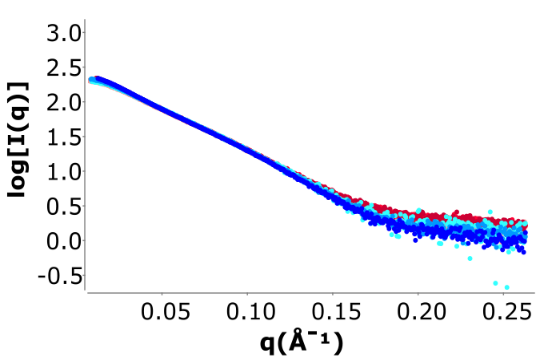

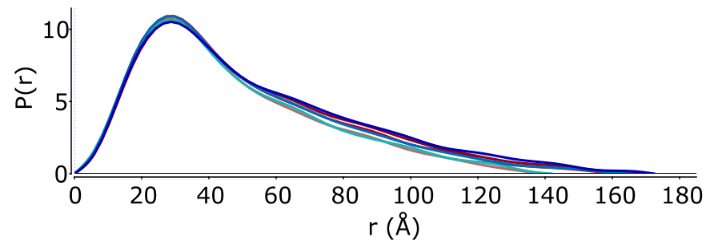


**Figure S5.** The best ANXA11 conformational ensembles selected by the program GAJOE aligned to the C-terminus. A) apo ANXA11 B) holo ANXA11. The flexible N-terminal regions may be in the proximity of the core than fully extended.


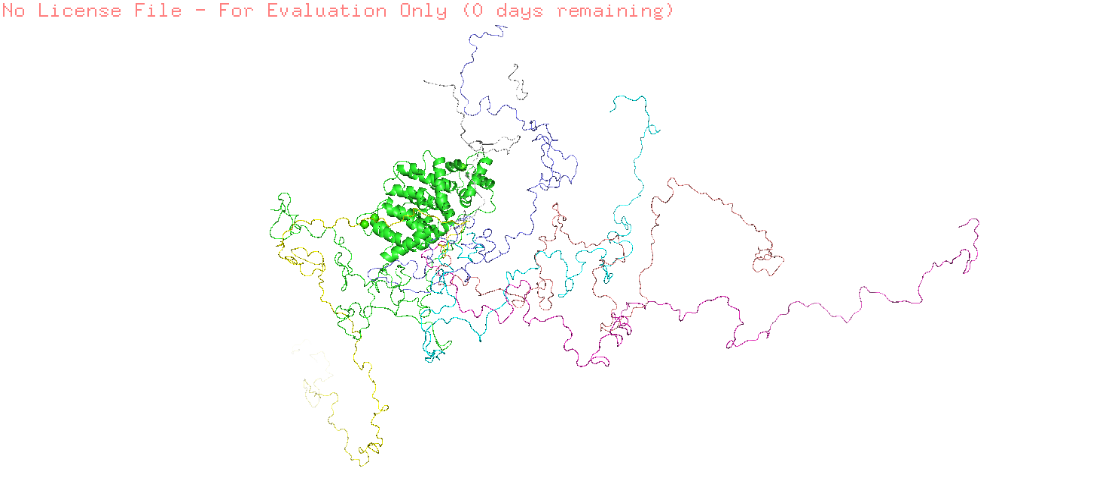

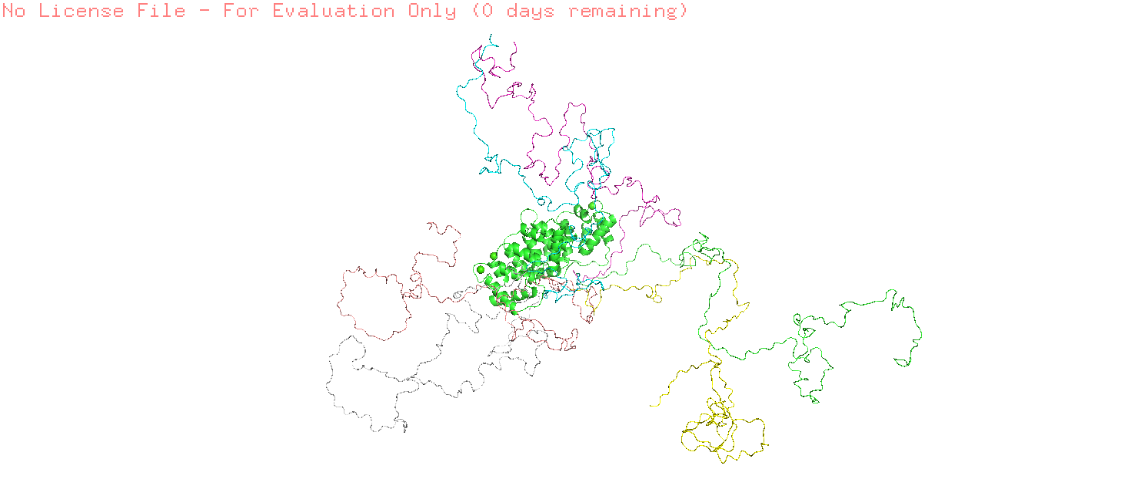

Supplement: Supplementary file 1 [file DataSheet1.docx]
